# Analysis of nosocomial bacteremia in an ICU during 16 months

**DOI:** 10.1186/cc9630

**Published:** 2011-03-11

**Authors:** L Cachafeiro, C Soriano, J Figueira, J Manzanares, J Camacho, M Jimenez Lendinez

**Affiliations:** 1 Hospital la Paz, Spain, Spain

## Introduction

The aim of our study is to evaluate the mortality, clinical impact and causative microorganisms of nosocomial bacteremia in the ICU of a tertiary university hospital.

## Methods

A prospective observational study in a 20-bed medical/surgical ICU, during a 16-month period. We included all patients admitted to the ICU >24 hours, excluding patients with acute coronary disease, from February 2009 to June 2010. We collected all episodes of bacteremia occurring in patients, demographics and epidemiological data, clinical impact, overall hospital mortality, ICU mortality and mortality related to bacteremia. Bacteremia type (primary, secondary, or connected to the catheter), microbiologic agents and empirical antibiotic therapy used.

## Results

A total of 1,112 patients were admitted to the ICU from February 2009 to June 2010. During this period, 63 nosocomial bacteremias were diagnosed in 45 patients, which represented 4% from the total admissions. The median age was 52 ± 16. Sixty-four percent were male. The median APACHE II score was 24 ± 9 versus 16 of all patients admitted during this period in the ICU (*P *< 0.05). The average stay of patients with bacteremia was 39 ± 25 versus 8 days of all patients (*P *< 0.01). Seventy-two percent of patients with bacteremia developed septic shock. The type of bacteremia: primary 35%: bacteremia/100 patients rate: 1.98; secondary 65%: bacteremia/100 patients rate: 3.68 (respiratory 25%, abdominal 19%, urinary 5%, skin 5%, CNS 2%, catheter 9%: bacteremias/1,000 VCC rate: 0.8). Seventy-eight percent were multidrug-resistant microorganisms. Mortality of patients admitted was 16% versus 40% overall mortality in patients with bacteremia (*P *< 0.01). Bacteremia was the direct cause of death of the patient in 27% of cases. Mortality with adequate empirical treatment was 8.2% versus 52% with inadequate treatment (*P *< 0.01). No patient died of bacteremia drug-sensitive organisms.

## Conclusions

Nosocomial bloodstream infections in the ICU make a major impact, with a high percentage of patients with septic shock, high morbi-mortality and hospital stay. Multidrug-resistant microorganisms played an important role in these results. It is necessary to optimize the control measures of the RBC and other devices, minimizing the multidrug-resistant microorganisms as well as empirical treatment protocols with broad-spectrum antibiotics.

## References

[B1] Burke JP Infection control N Engl J Med 2003 348 651 655 10.1056/NEJMhpr020557 12584377

